# Thermodynamic scaling behavior in genechips

**DOI:** 10.1186/1471-2105-10-3

**Published:** 2009-01-06

**Authors:** Alessandro Ferrantini, Joke Allemeersch, Paul Van Hummelen, Enrico Carlon

**Affiliations:** 1 Institute for Theoretical Physics, Katholieke Universiteit Leuven, Celestijnenlaan 200D, B-3000 Leuven, Belgium; 2 VIB MicroArray Facility, Herestraat 49, B-3000 Leuven, Belgium

## Abstract

**Background:**

Affymetrix Genechips are characterized by probe pairs, a perfect match (PM) and a mismatch (MM) probe differing by a single nucleotide. Most of the data preprocessing algorithms neglect MM signals, as it was shown that MMs cannot be used as estimators of the non-specific hybridization as originally proposed by Affymetrix. The aim of this paper is to study in detail on a large number of experiments the behavior of the average PM/MM ratio. This is taken as an indicator of the quality of the hybridization and, when compared between different chip series, of the quality of the chip design.

**Results:**

About 250 different GeneChip hybridizations performed at the VIB Microarray Facility for *Homo sapiens*, *Drosophila melanogaster*, and *Arabidopsis thaliana *were analyzed. The investigation of such a large set of data from the same source minimizes systematic experimental variations that may arise from differences in protocols or from different laboratories. The PM/MM ratios are derived theoretically from thermodynamic laws and a link is made with the sequence of PM and MM probe, more specifically with their central nucleotide triplets.

**Conclusion:**

The PM/MM ratios subdivided according to the different central nucleotides triplets follow qualitatively those deduced from the hybridization free energies in solution. It is shown also that the PM and MM histograms are related by a simple scale transformation, in agreement with what is to be expected from hybridization thermodynamics. Different quantitative behavior is observed on the different chip organisms analyzed, suggesting that some organism chips have superior probe design compared to others.

## Background

In Affymetrix Genechips the DNA sequences attached to the chip surface are synthesized *in situ *by photolitography [1]. The technology is limited to 25-mers, which are rather short oligos to provide a reliable measurement of the abundance of the complementary sequence in solution. Fortunately the same technology allows to place many more different sequences on a same given surface area compared to spotted arrays. Hence the loss of specificity due to the short length of the oligos can be compensated by the large number of sequences. For expression measurements typically a given mRNA transcript is interrogated by a *probe set *constituted by 10 to 20 probe pairs. Each probe pair is composed of two short oligonucleotides (25 pb). One matches with a part of the given mRNA transcript and it is called *perfect match *(PM). The mismatch probe (MM) has the same sequence, except at position 13, i.e. exactly in the middle nucleotide of the 25-mer. We use here the standard nomenclature of *probes *for the single stranded sequences attached to the chip surface and of *targets *for the transcript in solution.

The original purpose of the PM/MM design was to use the differences *I*_*PM *_- *I*_*MM *_as estimators of the probe specific binding. This is however problematic for two reasons: 1) For a fair amount of probes *I*_*MM *_> *I*_*PM *_(bright mismatches, which can occur in 30% of the probes in some chips, as discussed by [2]) and 2) in spike-in experiments, in which some target sequences are added in solution at well-known concentrations, *I*_*MM *_increases with increasing target concentration. Concerning point 1), Binder and Preibisch [3] showed that bright MM are predominantly observed at low intensities, i.e. for weakly expressed genes. Hence their origin is most likely correlated to non-specific binding favoring MM compared to PM. Point 2) reveals a more fundamental problem: the increase of the MM signal following an increase of target concentration implies that hybridization of the almost complementary sequence to a target cannot be neglected. Therefore MMs cannot be used as estimators of non-specific hybridization.

In this paper we demonstrate that MMs still hold some valuable information. In particular, we will focus on the average ratio of background subtracted PM and MM intensities. On general grounds, one expects that this ratio is constant and independent of the details of the type of experiment (e.g., tissue or organism analyzed). Our study focuses on three organisms: *H. sapiens*, *A. thaliana*, and *D. melanogaster*, for which in total more than 250 chips were analyzed, by means of the Langscal package presented here. The data were obtained over a period of about 4 years at the Microarray Facility of the Flemish Institute for Biotechnology (VIB). By investigating a large set of data from the same source we minimize the systematic variations between experiments that may arise from either slight differences in protocols or from different laboratories. The results presented here extend the findings of a previous study on some randomly selected experiments from the Gene Expression Omnibus server ([4], ). Indeed, the data from the same laboratory show a much more coherent behavior compared to the previous analysis, although systematic deviations for different organisms are clearly observed.

The Langmuir model, which has been used also in previous studies of Affymetrix Genechips (see e.g. [2,5]), predicts that the background subtracted PM/MM ratio depends only on the difference in chemical affinities (ΔΔ*G *in Eq. (2)) between the transcript sequence binding to a perfect matching probe and the same sequence binding to a probe with an internal mismatch. This quantity is related to the hybridization chemistry and depends only on the probe sequence. Therefore the ratio is expected to be independent on the biological details, as the organism or tissue analyzed. Our study shows that this is only partially true. Through a scaling analysis of PM and MM histograms we show that experiments in same organism have a quite stable PM/MM ratios. However, when different chips are compared, this ratio differs considerably.

We argue that these differences are due to the chip design. In the three organisms analyzed we found that the human chip had the smallest PM/MM ratio, and this ratio varies in two different human chips considered: HGU95 and HGU133, where the latter is based on a more recently annotated genome. In addition, the analysis of the PM/MM ratio and thermodynamic scaling can be used as a quality control for a global check of the performance of an experiment. Langscal is an R-package designed for this kind of analysis [6]. It computes average PM/MM ratios and investigates scaling properties of intensity histograms. This information provides insights about the agreement of the experimental data with hybridization thermodynamics and also a global check of the quality of an experiment. Langscal is freely available at .

## Results and discussion

### Global histograms

**Figure 1:**
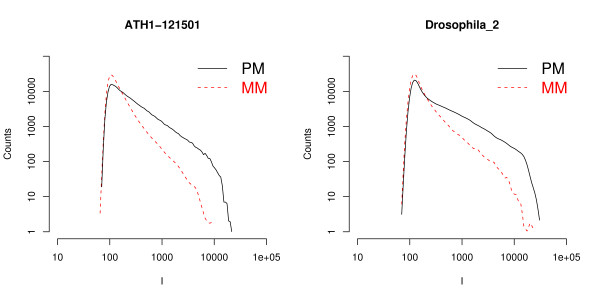
**PM and MM intensity histograms**. Histograms of the intensities of PM and MM for two experiments on *Arabidopsis thaliana *and *Drosophila melanogaster*. These histograms show very similar features: a common behavior at low intensities followed by a split of the PM and MM histograms at higher intensities.

In Fig. 1 we provide two examples of global histograms of the raw intensities of the PM and of the MM for a whole chip. The data are plotted in log-log scale, and the intensity values span almost three orders of magnitude. In the low intensity region the histograms present a bell-like shape, which is mainly expression of background signal and PM and MM histograms overlap. At higher intensities, as it could be expected, there are less MM probes compared to PM. Similar types of histograms are found in the other experiments analyzed, although there are some variations from experiment to experiment and from organism to organism (see Additional file 1 for additional examples). The sudden drop of the histogram at intensities close to *I *≈ 15, 000 can be understood from the Langmuir isotherm (Eq. (1) in Methods). This isotherm links the intensity to the target concentration *c *and binding affinity Δ*G*. From Eq. (1) one finds that at sufficiently high *c and *Δ*G *the signal saturates at *I *= *I*_0 _+ *A *≈ *A *(*I*_0 _in Eq. (1) is the background level, and it is typically much lower than the saturation value, hence *I*_0 _≪ *A*). At saturation all available probes are hybridized and the signal can no longer increase. From the two plots in the figure a rough estimate of the saturation threshold is *A *≈ 15, 000 for the *A. thaliana *chip and *A *≈ 20, 000 for the *D. melanogaster *chip. Note that there are only a few hundred probes whose intensities are close to the saturation value (see Fig. 1). Hence, when analyzing the average PM/MM ratio it is safe, for practical purposes, to neglect the denominator of Eq. (1), as mentioned in Methods.

There has been some discussion about the variability of the saturation threshold (*A *in Eq. (1)) for different probes [5] and about the differences in the PM and MM saturation, possibly due to post-hybridization effects [7]. The sudden drop at high intensity in the histograms analyzed in our study, however, suggests a probe independent threshold as expected from the Langmuir isotherm. In addition, the analysis of Affymetrix spike-in data [8] shows a good agreement with the Langmuir model with a unique probe-independent saturation threshold *A*. It is difficult to assign a threshold to the MM histograms as they decay rapidly at high intensity: no MM probes have an intensity above 10, 000 in the *A. thaliana *chip of Fig. 1, while just a few MM probes are above this value in *D. melanogaster*. However the data are not inconsistent with a common saturation value for PM and MM histograms.

### Rescaling

After having presented some global features of the histograms we proceed with the illustration of the results of the computations of the PM/MM ratios, which are based on Eq. (5) and (6) in Methods. These equations, deduced from the Langmuir model far from saturation, link the PM and MM histograms to each other by a simple scale transformation. The link holds for probes with a similar type of mismatch. As explained in Methods, this boils down to separate the PM/MM pairs to those having a common central triplet. There are in total 64 different triplets, which we label with the symbol *α*. The starting point of the analysis is the calculation of the background level *I*_0_.

The Langscal package estimates *I*_0 _as the crossing point between the global PM and MM histograms of intensities, as it is shown in Fig. 1. The user can eventually refine this estimate by providing his own value of *I*_0_. Afterwards the 64 ratios *a*_*α *_are calculated from the median of the background subtracted PM/MM ratios, following Eq. (5).

**Figure 2:**
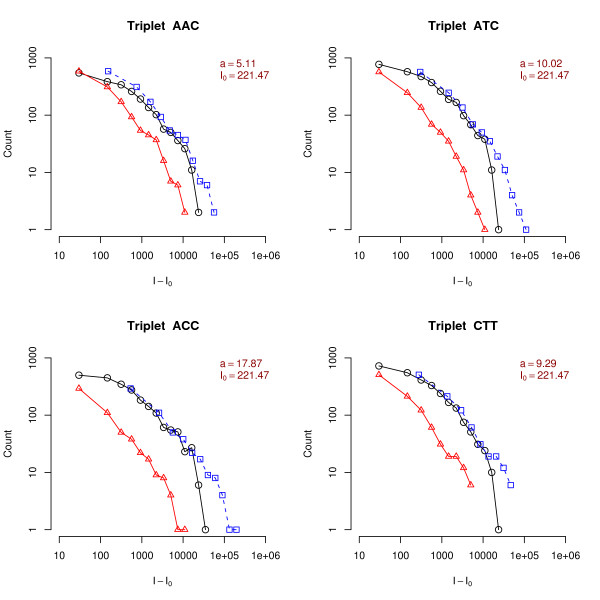
**Rescaling of central triplet histograms**. Histograms of the background subtracted intensities of PM (circles) and MM (triangles) for a *D. melanogaster *chip. The probes are now grouped according to their central triplet; only 8 out of 64 cases are shown. The dashed lines (squares) are obtained by multiplying the MM histogram by a factor *a*_*α*_, which results in a horizontal shift of the background corrected intensity histogram when plotted in log-log scale. The overlap between the PM and rescaled MM histograms is a proof of the validity of Eq. (6). We recall that the central triplet sequence is that of the PM probe, hence, say, a ACC triplet corresponds to a mismatch of GG type.

**Figure 3:**
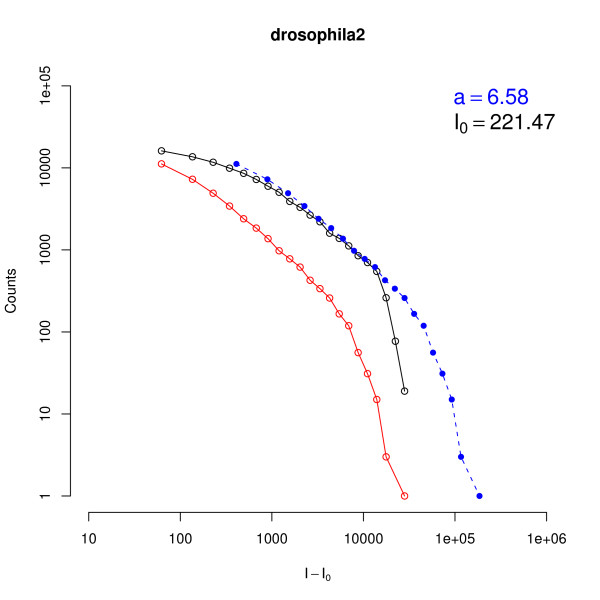
**Rescaling of global histograms**. Histograms of the background global subtracted intensities of PM (black circles)  and MM (red circles) for a *D. melanogaster* chip. The dashed line is obtained by multiplying by a factor *a*, calculated as indicated in Eq. (5), the MM histogram. Note that the overlap of the solid and dashed curves is good except for high intensity values where saturation affects the signal in a significant way.

In Fig. 2 we report the histograms of the background subtracted PM and MM intensities for different types of triplets for an experiment on the *D. melanogaster *chip. The triplets shown in the figure correspond to different mismatches and different flanking nucleotides. The values of *a*_*α *_are shown on top of each graph. They vary from 3.75 for a CGT triplet up to 13.2 for an ACC triplet. These differences are related to the different values of ΔΔ*G *associated to each mismatch type. Note that for AA or GG mismatches *a*_*α *_'s are usually higher compared to TU or CC mismatches (in Fig. 3 triplets ACC or CCT correspond to GG mismatches, while triplets AGC or CGT to CC mismatches). This can be understood from steric effects: purines have a double aromatic ring and are more bulky than single ring pyrimidines, hence a purine-purine mismatch (as AA or GG) is sterically unfavored compared to a mismatch pyrimidine-pyrimidine one [2]. Additional file 1 shows a large collection of the PM/MM ratios *a*_*α *_for different triplets. The analysis of the large number of independent experiments on the three organisms shows a trend of hierarchies for the different triplets which is quite reproducible and it is also qualitatively consistent with the experimentally measured parameters ΔΔ*G*, as reported previously [4]. Note however that, quantitatively, the *a*_*α *_obtained from Eq. (5) using the nearest neighbor model ΔΔ*G*'s as obtained in the experiments of [9,10] is in the range 40 ≲ *a*_*α *_≲ 3000, while the typical values in microarrays experiments are 1 ≲ *a*_*α *_≲ 20. The origin of these differences will be discussed in the next section.

Figure 2 also shows the MM histograms in which the background subtracted intensity is multiplied by a factor *α*_*s*_, which corresponds to a horizontal shift in the log-log scale. The fact that the PM and shifted MM histograms overlap is a proof of the validity of Eq. (6), which shows that the experimental data are consistent with the linearized Langmuir model. The accuracy of the overlap can be interpreted as an estimator of the quality of the hybridization and its consistency with equilibrium thermodynamics. Sometimes the histogram rescaling may be not as good as in Fig. 2, as shown in few a examples in Additional file 1. The poor overlap is a sign of an inconsistency with the scaling form predicted from Eq. (6) and can have different origins, which will be discussed in the next section.

To summarize the triplet analysis with a single chip parameter, we also performed the rescaling of Eq. (6) for the histogram over the whole chip, i.e. ignoring the differences between triplets. The global factor *a *is then found by requiring the overlap between PM and shifted MM histograms. An example of such global rescaling is shown in Fig. 3 for an experiment on *D. melanogaster*. In this case the average ratio between the background subtracted PM and MM intensities is *a *≃ 6.5. Note that the histograms are smoother than those shown in Fig. 2, as they are extended to the intensities of the whole chip. The rescaled MM histogram (dashed line) overlaps well with the PM histogram in most of the intensity range. A deviation is observed in the high intensities regime where the PM histogram drops and splits from the rescaled MM histogram. This behavior is described by the denominator in the Langmuir isotherm in Eq. (1). As mentioned before, the histogram rescaling (Eq. (6)) is only valid not too close to the saturation level, a regime in which the denominator of Eq. (1) can be neglected. As also stated before, the saturation regime only involves a few hundred PM probes, a small fraction of the total number of probes.

**Figure 4:**
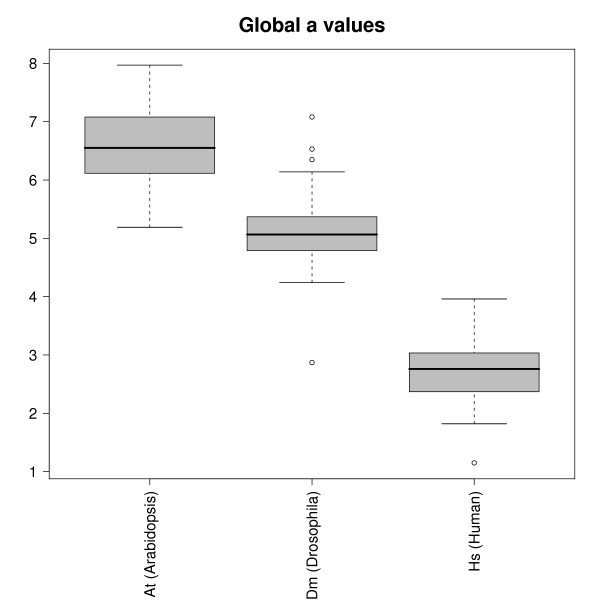
**Global PM/MM ratio for different organisms**. Boxplot of the global PM/MM ratio of all the experiments analyzed sorted by chip/organism type. Hybridization thermodynamics would predict a constant ratio. The analysis, however, shows that this ratio depends on the organism. Within a given species PM/MM ratios are rather constant, they do not seem to depend on tissue analyzed or other biological conditions. Very few outliers are found within each organism (see discussion in the text).

Figure 4 shows boxplots for the global *a *parameters calculated from the rescaling of the global intensity histograms as shown in Fig. 3. The data are collected for each organism separately. Within each organism the value of *a *is quite constant, while we observe some systematic variation between the different organisms. *A. thaliana *has the highest PM/MM global ratios with an average value of *a *≃ 6.5, while for *D. melanogaster a *≃ 5. The human chip (HGU133a) shows the smallest ratios (i.e., *a *≃ 2.5).

Very few outliers are observed outside the boxplot range. One example is a human chip with *a *≃ 1, a value which implies overlapping PM and MM histograms. This is a chip with an anomalously high intensity background signal and it is as well flagged as unsatisfactory hybridization based on the Affymetrix guidelines and from image inspection (, see Table 1 in the Additional file 1).

**Figure 5:**
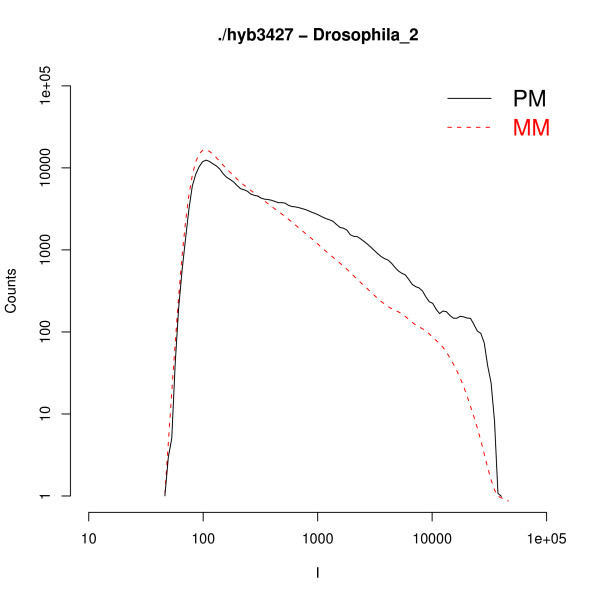
**PM and MM histograms for a D. Melanogaster chip**. PM and MM histograms of a *D. melanogaster *experiment with a big stain on it. The plot already shows that both histograms are unusually close to each other. This anomalous behavior yields unavoidably low PM/MM background subtracted ratios: e.g. in this case the analysis gives a global *a *= 2.87, instead of the typical *a *≃ 5 found in the Drosophila chips.

Other outliers are found in the *Drosophila *hybridizations. One chip has an anomalously low PM/MM ratio *a *≃ 3, instead of the typical *a *≃ 5 for *Drosophila *chips. Figure 5 shows the global PM and MM histograms for this anomalous chip. From a comparison with the *Drosophila *chip histograms in Fig. 1 one can clearly see a difference between the two cases. Indeed, the low value of *a *can be also visualized from the closeness of PM and MM histograms of Fig. 5. This chip is also flagged as hybridization of insufficient quality according to the Affymetrix guidelines (see Additional file 1, Table 1). An analysis of the fluorescence image in the whole chip shows a clear stain on the chip (see Additional file 1, Figure 1). The stain is not responsible for any clear high background value, but the anomaly is still well pointed out by the low value of the PM/MM ratio.

Three other *Drosophila *chips were found to have a substantially bigger value of the PM/MM ratio, compared to the average *Drosophila *value *a *≃ 5. The analysis of the standard quality checks according to the Affymetrix guidelines for these three chips does not show any kind of low quality behavior. These chips come all from the same experimental batch, which suggests that the sample extraction, preparation and amplification may influence the value of *a*. In general (see Conclusions) one expects that a large value of the PM/MM ratio is a signature of good hybridization quality. Thus these 3 *Drosophila *chips with high *a*'s are probably the result of an experiment of particularly good quality.

**Figure 6:**
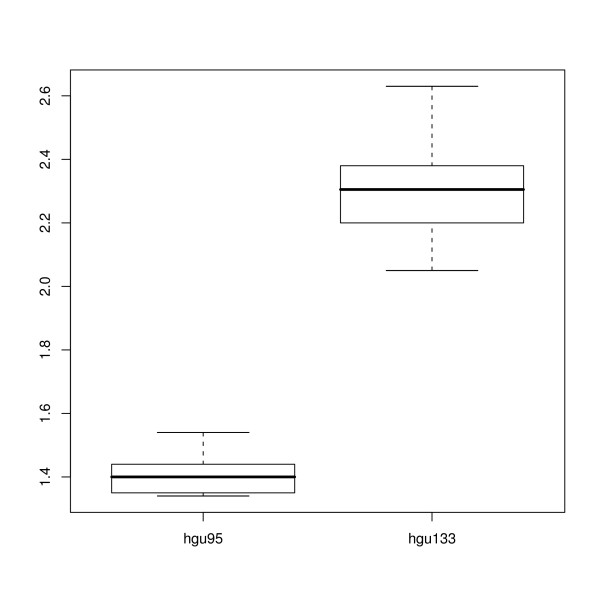
**PM/MM ratios for HGU95 and HGU133 chipsets**. Boxplot of the global PM/MM ratio of human chips sorted by type (HG-U95 and HG-U133). The data are taken from the spike in experiments available on the Affymetrix website.

In order to assess the influence of a chipset design on hybridizations within the same organism, we plotted in Fig. 6 the values of *a *for two sets of experiments downloadable from the Affymetrix web site. These are the Latin square data, in which few genes are spiked-in at known concentration into a complex mixture. The two experiments were performed on the older chipset HGU95 and on the more recent HGU133. They show a quite different value of the parameter *a*, implying that the chip design may influence PM/MM ratio. Note also that the value of *a *≃ 2.5 is the same in the HGU133 chipsets analyzed at the VIB Microarray Facility (Fig. 4) and from Affymetrix spike-in data set (Fig 6).

## Conclusion

One of the first concerns raised by DNA microarrays was that of quality control and check of the reliability of the technology. A microarray experiment consists of many biochemical steps, which contain each many potential sources of variations, influencing the overall performance of the experiment. Therefore it is extremely important to have tools for the quality assessment in order to be able to eventually discard experiments in which the quality does not meet certain standards. Some of these tools indeed exist, as e.g. the Bioconductor package affyPLM [11,12], which is based on probe level data analysis, and simpleAffy [13], which allows to assess the Affymetrix quality standards.

This paper presents another tool for the quality control of hybridization in Affymetrix Genechips, which relies on the analysis of PM and MM histograms. Through this analysis one can check the agreement of the data with the hybridization thermodynamics, which predicts that the intensity is linked to the target concentration and hybridization free energy according to the Langmuir isotherm of Eq. (1). A consequence of the Langmuir isotherm is that the background subtracted PM/MM ratio of Eq. (2) is *universal*, i.e. it should depend only on the nature of the nucleotides neighboring the mismatch site and not on the biological details of the experiment, as organism or tissue investigated. The Langmuir isotherm predicts that the histograms of background subtracted PM and MM intensities should scale on top of each other when a shift of the MM intensity scale is applied.

The Langscal package presented here has been designed with the purpose of checking the validity of these hypothesis and to determine the PM/MM ratios for all different mismatches which are organized in triplets. In agreement with previous results [4] the values of *a*_*α *_follow the hierarchy expected from experimental studies involving DNA/RNA hybridization in solution [9,10]. The previous study [4], done on a series of randomly selected experiments, showed a large variability from experiment to experiment of the parameters *a*_*α*_. In the data presented here, and when focusing on the same organism, one finds stable values of the *a*_*α*_, except for a few outliers. This shows that the different protocols used, as hybridization characteristics or the stringency of washing may influence considerably the PM/MM ratio.

Quantitatively there is, however, a difference of at least one order of magnitude between the values of *a*_*α *_deduced from hybridization studies in solution and those which are actually measured on the microarray.

There are several reasons for these differences. For instance, hybridization in solution is different from microarray hybridization which occurs in the vicinity of a solid surface and the value of ΔΔ*G *obtained from hybridization in solution [9] may not adequately describe hybridization in a chip. The fact that *a*_*α *_is lower than its solution counterpart is in agreement with the idea that hybridization is impeded by the vicinity of the surface, leading to an overall lowering of Δ*G*. A second effect is that target sequences are typically fragmented before hybridization. Such fragmentation may lead to hybridization to the MM probe of two fragments that skip the 13th mismatching nucleotide. If such events are frequent, then the estimate of Eq. (2), which links the PM/MM ratio to ΔΔ*G *is clearly inadequate. Irrespectively of the origin of these differences, one could anyhow take the parameters *a*_*α *_as measurements of the hybridization quality.

High values for *a*_*α *_suggest that the hybridization took place in conditions close to the thermodynamic equilibrium in which both strands are in solution.

In order to summarize the analysis with a single quality parameter we have used the global PM/MM ratio over the whole chip, *a*, where no distinction is made between triplets. The boxplot of the *a *measured in different experiments for the three different organisms analyzed is shown in Fig. 4. The first surprising result is that the global PM/MM ratio depends on the organism studied, while hybridization thermodynamics (see Eq.(1) and (2)) suggests that it should only depend on the chemistry of the hybridization process and not on any other biological details as the tissue, organism, or the biological conditions used. This is however only true in the case of an optimal probe design in which stringent criteria for probe uniqueness would ensure a dominant specific hybridization with the desired target sequence. When these criteria are not met, other target sequences may bind significantly to the probe and they can do so more efficiently to a MM than to a PM and therefore modify the PM/MM ratio.

Using *a *as a measurement for the hybridization quality and according to the large amount of data analyzed, we found that the best performance (highest *a *value) is that of *A. thaliana *chips, with an average ratio of about 6.5, while the poorest is that of the human chips (average *a *≃ 2.5). It is maybe not surprising to find the lowest values for *a *for the human chips. Within the three organisms investigated, the human genome is by far the most complex one in terms, for instance, of alternative splicing events or of Single Nucleotide Polymorphisms. These complications may lead to PM/MM pairs which do not perform as expected from hybridization thermodynamics.

The correlation between the average ratio *a *and the probe design can be clearly seen in Fig. 6. This shows an increase from *a *≃ 1.4 for HGU95 to *a *≃ 2.3 for HGU133. The difference between these two human chipsets is that the HGU133 is more recent and has a more up to date annotation compared to the older chipset HGU95. An improvement in the chip design leads thus to an increase in the parameter *a*, consistent with the idea that this parameter can be used as a measure of the quality of the hybridization.

There are several possible problems in an experiment as excessive target fragmentation or sample contamination. In addition, a chip can contain annotation errors, which could lead for instance for a MM sequence to be perfect matching to other transcripts. These effects, which are not considered in the present analysis, would typically lead to lower the intensity differences between PM and MM pairs hence to a lower than average PM/MM ratio. When comparing experiments from different laboratories one may find a wide range of values for the average PM/MM ratio, because the experimental protocols and sample preparation can considerably influence the ratio. Nonetheless, a systematic study of this ratio in different chip organisms and experimental designs could be very useful for a better understanding of the origin of the lab-to-lab variability, which is a weakness to current microarrays.

In conclusion our work started from some theoretical predictions on the scaling behavior of PM and MM histograms. On the view of the fact that the analysis is based on a simple model, with only one adjustable parameter (the average background intensity value), we can conclude that the scaling hypothesis is satisfactorily verified in most of the experiments. Also the hierarchy of values of triplet-dependent PM/MM ratios follows the same inequalities as expected from equilibrium thermodynamics [4]. These ratios are however rather constant only for experiments within the same chip, and not for different organisms chips. This result is at odds with thermodynamics and it is probably due to problems with the chip design. So far the analysis of the shape of microarray intensity histograms has received little attention in the literature. We think that such analysis provides interesting insights on the global performance of an experiment and on a better understanding to the relationship between PM and MM. The Langscal package, designed to perform such an analysis, is useful for a global quality check, together with other quality control methods [11-13] prior to expression level estimation.

## Methods

### Langmuir hybridization thermodynamics

To understand the PM/MM relationship and to check its consistency with hybridization thermodynamics, a software package has been developed. This software relies on the Langmuir model, which was invoked in a recent work to explain hybridization behavior in DNA microarrays [2,5,8,14]. The Langmuir model links the measured fluorescent intensity *I*_*η *_from a given PM or MM feature (*η *= *PM*, *MM*) to the target concentration *c *as:

(1) $$I_{\eta }=I_{0}+\frac{Ace^{\Delta G_{\eta }/RT}}{1+ce^{\Delta G_{\eta }/RT}}$$

where *I*_0 _is the background intensity level, that one would measure in absence of a target (limit *c *→ 0).

The factor *A *is a proportionality constant, *R *the gas constant, and *T *the temperature. The hybridization free energy Δ*G*_*η*_, which is sequence-dependent, measures the affinity of the target-probe binding. Note that the specific bindings to a PM or MM probe are treated on equal footing, but with different hybridization free energies Δ*G*_*PM *_and Δ*G*_*MM*_. DNA hybridization thermodynamics [15] predicts the highest affinity for perfect matching hybridization compared to hybridization with one or more internal mismatches, hence Δ*G*_*PM *_> Δ*G*_*MM*_.

We assume that in Eq. (1) the PM and MM background intensities are approximately equal (same *I*_0_), which is indeed the case for a fair fraction of probes in Affymetrix spike-in experiments [16]. Moreover, for the large majority of the probes, the denominator in Eq. (1) can be neglected, as we will show explicitly below. Using these approximations one finds from Eq. (1) for a given PM/MM probe pair:

(2) $$a\equiv \frac{I_{PM}-I_{0}}{I_{MM}-I_{0}}=e^{\Delta \Delta G/RT}$$

where ΔΔ*G *= Δ*G*_*PM *_- Δ*G*_*MM *_is the difference between the hybridization free energies of a PM and a MM. Note that in the ratio of Eq. (2) the target concentration c (expression level) cancels out. Hence, according to the model, the background subtracted ratio for a given PM/MM pair should be constant across different experiments, as this quantity depends only on ΔΔ*G*, which in turn depends only on the sequence composition. Individual PM and MM signals may of course vary because of changes of expression levels *c *in different experiments, as it is clear from Eq. (1). Actually, as discussed below, ΔΔ*G *is expected to be dependent only on the central nucleotide triplet. A consequence of the hybridization thermodynamics is that the ratio of Eq. (2) is constant throughout experiments for PM/MM pairs with the same central nucleotide triplet.

The use of a constant background value *I*_0 _is an approximation. Algorithms for the computations of the expression level typically use background estimators which are probe dependent (see e.g. [12]). In the present work, however Equation (2) will be tested on an average of typically several hundred probe pairs sharing the same central nucleotide triplet. As robust estimator the median over individual ratios will be taken. We verified that a change of 10% in Eq. (2) leads to a change of about 5% of the average ratios in Fig. 4, while a smaller change of 5% of the background would lead to a change of less than 2%. We conclude that results on average ratios do not critically depend on the choice of background value. In order to keep the analysis simple a single constant background value is chosen.

### Triplets

The Nearest Neighbor Model provides a method for the calculation of the hybridization free energy Δ*G *for a given sequence (see e.g. [15]). It is based on the assumption that the stability of each base pair depends on the identity and orientation of the adjacent base pairs. For RNA/DNA interactions, which are of relevance in Affymetrix expression arrays (the target sequences are RNAs), the Nearest Neighbor parameters were experimentally determined by [9]. There are 16 of such parameters which account for all possible combinations of neighboring pairs. For instance for a given sequence stretch of 5 nucleotides, within the Nearest Neighbor Model one calculates Δ*G *as follows:

(3) $$\Delta G\left(\begin{array}{c} \text{dATGAC}\\ \text{rUACUG} \end{array}\right)=\Delta G\left(\begin{array}{c} \text{dAT}\\ \text{rUA} \end{array}\right)+\Delta G\left(\begin{array}{c} \text{dTG}\\ \text{rAC} \end{array}\right)+\Delta G\left(\begin{array}{c} \text{dGA}\\ \text{rCU} \end{array}\right)+\Delta G\left(\begin{array}{c} \text{dAC}\\ \text{rUG} \end{array}\right)+\Delta G_{\text{in}}$$

where the parameters $\Delta G\left(\begin{array}{c} \text{d}\bar{\text{x}}\bar{\text{y}}\\ \text{rxy} \end{array}\right)$ are tabulated and where Δ*G*_in _is the helix initiation parameter, which depends on the nature of the nucleotides at the double strand edges. [10] also considered the case of single internal MMs. The parameters for mismatches are dependent on the type of mismatch and also on the identity of the two flanking nucleotides. In Affymetrix Genechips the MM sequence is constructed with the substitution rule A ↔ T and C ↔ G. As an example of calculation of the value of ΔΔ*G *within the nearest neighbor model we consider a PM probe with central nucleotides CGC and consequently of type CCC in the MM probe. One finds:

(4) $$\Delta \Delta G=\Delta G\left(\begin{array}{c} \text{d…CGC…}\\ \text{r…GCG…} \end{array}\right)-\Delta G\left(\begin{array}{c} \text{d…C}\underline{\text{C}}\text{C…}\\ \text{r…G}\underline{\text{C}}\text{G…} \end{array}\right)$$

where d and r indicate the DNA probe and RNA target strands, respectively. The dots indicate the flanking nucleotides: as these are identical in the two stretches of the sequence they give equal contribution to both Δ*G*'s and hence cancel out in ΔΔ*G*. The result is thus that ΔΔ*G *depends only on the identity of the central nucleotide triplet.

The substitution rule A ↔ T and C ↔ G implies that 4 types of mismatches can be generated (i.e., AA, CC, GG, and TU). Taking into account the two flanking nucleotides, one ends up with 64 possible central triplets. Using Eq. (4) and the free energies given in [9] and [10] one can find estimates for ΔΔ*G *of 8 triplets, as experimental values are available only for these. From the given ΔΔ*G*'s and setting *T *= 45°*C *in Eq. (2), one finds typical values of *a *in the range of 40 to 3, 000. These values are obtained from estimates of Δ*G *parameters for hybridizations in which both strands are in solution. They will be compared with actual PM/MM ratio determined from the analysis of the experimental data in the next section.

### Scaling of PM and MM histograms

As ΔΔ*G *depends on the nature of the central triplet, we consider next the analysis of the ratio of Eq. (2) for each triplet separately. We label them with *α *= {AAA, AAC, AAG, ..., TTT} and define:

(5) $$a_{\alpha }={\langle \frac{I_{PM}-I_{0}}{I_{MM}-I_{0}}\rangle }_{\alpha }$$

where ⟨⟩_*α *_denotes the median over all probe pairs at fixed central triplet *α*. If the triplets are roughly equally frequent, a chip with 500, 000 sequences will approximately have about 8, 000 PM/MM pairs with a given *α*. In the previous equation we take for simplicity *I*_0 _constant in the whole chip and further restrict to probe pairs that satisfy the inequalities: *I*_*PM *_> *I*_0 _and *I*_*MM *_> *I*_0_.

As a check of the consistency of the values of the ratios computed as in Eq. (5), one can consider the relationship between PM and MM intensity histograms. Let $P_{\text{PM}}^{\alpha }$, $P_{\text{MM}}^{\alpha }$ denote the histograms of the intensities for a given triplet *α*. From Eq. (2) it follows that ([4]):

(6) $$P_{MM}^{\alpha }(I-I_{0})=P_{PM}^{\alpha }(a_{\alpha }(I-I_{0}))$$

In practice, one can check the validity of Eq. (6) by plotting the PM and MM histograms on the same graph, but with two different scales on the horizontal axis: the MM background subtracted intensities should be rescaled by a factor *a*_*α*_. The overlap of the two histograms rescaled in this way is a direct proof of the validity of the Langmuir model and also of the consistency of the experimental data with thermodynamics. The package Langscal produces these rescaling plots for all the 64 triplets.

Finally, to characterize the PM/MM ratios of one experiment with a single parameter, the rescaling of the histograms of Eq. (6) can also be extended to all intensities on the chip.

### The experiments analyzed

We demonstrate the usage of the Langscal package on a randomly chosen set of Affymetrix hybridizations, performed at the VIB MicroArray Facility. The data set comprises 91 Affymetrix chips of array design ATH1-121501, designed for *A. thaliana*, 48 hybridizations on the Drosophila_2 chip, and 135 hybridizations of HG-U133_Plus_2 chip design, for *H. sapiens*. The arrays were processed by different lab-technicians over a period of 4 years.

The procedures, including amplification, labeling, and fragmentation, were performed manually or were automated by a Biomek-3000 ArrayPlex work station (Beckman-Coulter, Analysis Belgium). However, the procedures followed a standardized protocol with possibly small variations due to changes in kits provided by Affymetrix or in enzymes or other ingredients. In general, total RNA was controlled for its integrity and purity using Agilent Bioanalyzer and Nanodrop spectrophotometer , respectively. Only RNA without signs of degradation or impurities (260/280 and 260/230 > 1.8) was used for further processes. Probes were prepared from 2–5 *μg *total RNA according to Affymetrix's guidelines. Briefly, from total RNA, poly-A RNA was reversed transcribed using a poly dT-T7 primer and labeled during a T7 *in-vitro *transcription reaction using the Affymetrix IVT Labeling Kit (Affymetrix, High Wycombe, UK). The probes were purified (GeneChip Sample Cleanup Module, Affymetrix, UK) and analyzed again for yield (30–120 *μg*) and purity (260/280 and 260/230 > 1.8). 20 *μg *from the resulting aRNA was fragmented with alkaline hydrolysis and resuspended with control spikes in 300 *μl *hybridization buffer (Eukaryotic Hybridization Control Kit, Affymetrix, High Wycombe, UK). The Genechips were hybridized in a rotisseri oven at 45°C and washed and stained in the GeneChip Fluidics Station-400 or -450 (Affymetrix, UK) using EukGE-WS2 protocol. Arrays were scanned with the GeneChip Scanner 3000 (Affymetrix, UK) and image analysis was performed in GCOS.

### Availability

The R-package Langscal which performs the above analysis is freely available at .

## Authors' contributions

AF and EC developed the theory of PM/MM scaling. AF and JA wrote the R-code for the data analysis and analyzed the data. PVH provided all experimental data and with EC supervised the analysis. AF, JA and EC wrote the paper. All authors have read and approved the final version of the manuscript.

## Supplementary Material

Additional file 1 **Langmuir scaling behavior in genechips – Supplementary online material**. This document presents data analysis results of additional data and, hence, provides results of a more extensive data set and a larger variety of gene chips. Click here for file
