# Supplementary material for: Thermodynamic scaling behavior in genechips
Source: BMC Bioinformatics. 2009 Jan 6;10:3. doi: 10.1186/1471-2105-10-3 (PMC2628882; doi:10.1186/1471-2105-10-3)
Supplement: Additional file 1 — Langmuir scaling behavior in genechips – Supplementary online material. This document presents data analysis results of additional data and, hence, provides results of a more extensive data set and a larger variety of gene chips. [file 1471-2105-10-3-S1.pdf]

Langmuir Scaling behavior in Genechips – Supplementary Online Material

A. Ferrantini, J. Allemeersch, P. Van Hummelen, and E. Carlon

June 2008

Additional PM and MM histograms

We present here some additional figures of histograms and ratios as obtained by the Langscal package for Arabidopsis (Fig. 1) Drosophila (Fig. 2) and Human (Fig. 3) chips. All of these have common features: the MM histograms decay faster at high intensities, compared to PM histograms. Notice, though, that for human chips the PM and MM histograms are much closer to each other. This is reflected in a lower value of the PM/MM ratio as discussed in the paper.

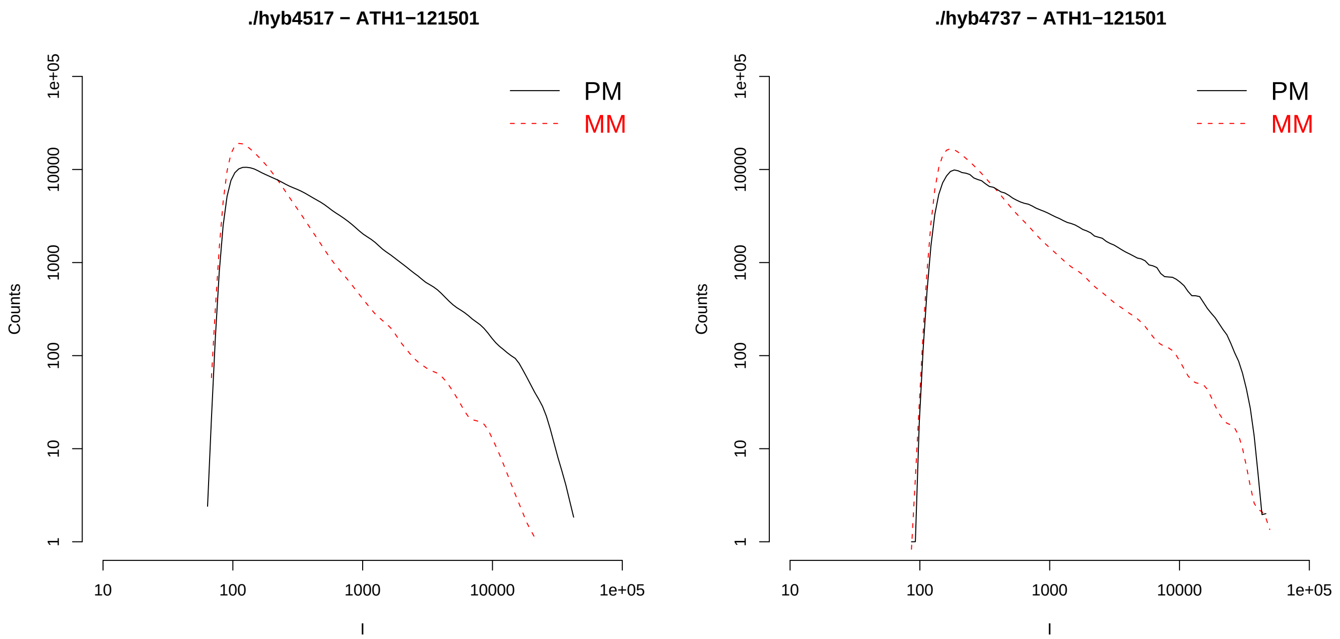

Figure 1: Global histograms of the intensities of PMs (black circles) and MMs (red triangles) for two experiments on *A. thaliana*.

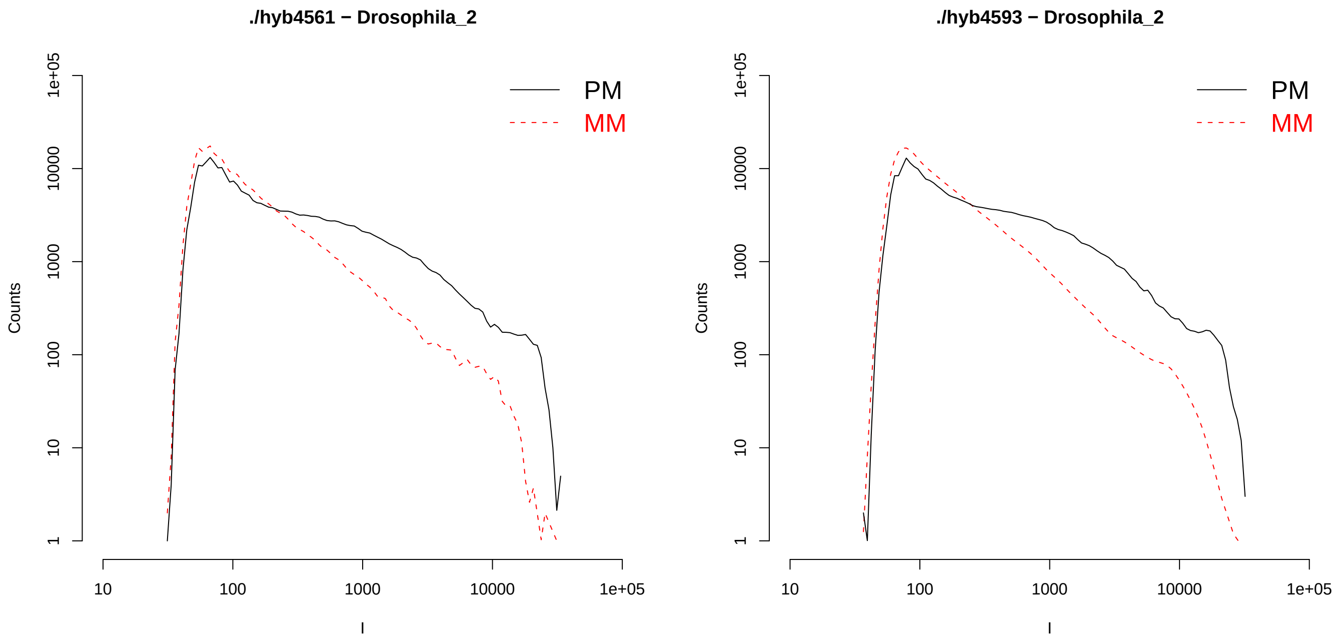

Figure 2: Global histograms of the intensities of PMs (black circles) and MMs (red triangles) for experiments on *D. melanogaster*.

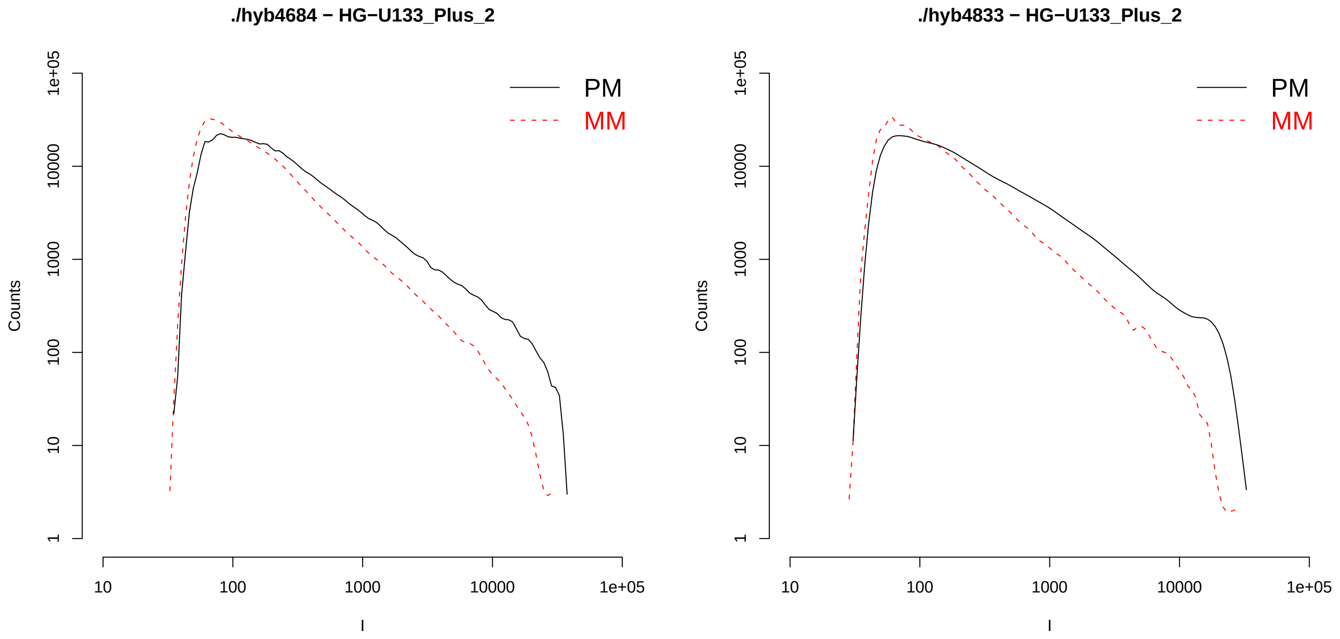

Figure 3: Global histograms of the intensities of PMs (black circles) and MMs (red triangles) for experiments on *H. sapiens*.

A mathematical consideration on global histograms

In this section we link the decay of the intensity to the distribution of gene expression levels. The starting point is the Langmuir isotherm:

$$I - I_0 = \frac{Ace^{\Delta G/RT}}{1 + ce^{\Delta G/RT}} \tag{1}$$

where we use the same notation as in the paper: I_0 the non-specific background, A a global constant, ΔG the hybridization free energy and T the temperature.

As only a small fraction of probes are close to saturation (see histograms in Figs 1, 2 and 3), when discussing global properties of the histogram we can neglect the denominator and approximate the isotherm as:

$$I - I_0 \simeq Ace^{\Delta G/RT} \tag{2}$$

which is the same approximation used throughout the paper.

We consider next the probability $p(c, \Delta G)$ to find a target with a hybridization free energy to its probe equal to ΔG and a concentration c. The histogram is related to the joint probability $p(c, \Delta G)$ as follows:

$$P(I - I_0) = \int\int p(c, \Delta G)\delta\left(I - I_0 - Ace^{\Delta G/RT}\right) d(\Delta G)\, dc \tag{3}$$

where $\delta(x)$ is the delta function. Note that this expression holds for PM and MM, the difference between the two being a different joint probability distribution $p(c, \Delta G)$.

It is reasonable to assume that the expression level c and ΔG (which is sequence dependent, see text) are uncorrelated so that the joint probability factorizes as $p(c, \Delta G) = r(c)q(\Delta G)$. Hence:

$$P(I - I_0) = \int\int r(c)q(\Delta G)\delta\left(I - I_0 - Ace^{\Delta G/RT}\right) d(\Delta G)\, dc \tag{4}$$

In order to infer the behavior of $P(I - I_0)$ we need to be able to characterize the probability distributions $p(c)$ and $q(\Delta G)$ which appear in Eq. (3). Fig. 4 shows the probability distribution for ΔG for PM and MM probes in a human chipset. Both distributions are well approximated by gaussians. Similar results were found in other chips analyzed. The mean value and spread of the gaussian varies from chip to chip.

In general the integral in Eq. (4) is not tractable for an arbitrary gene expression distribution $r(c)$. One solvable case is that of a distribution of power-law form, i.e. of the type:

$$r(c) = \frac{\alpha}{c^\gamma} \tag{5}$$

α being a constant. We recall a property of the delta function, namely if a function $f(x)$ has a single zero in x_0 then:

$$\delta(f(x)) = \frac{1}{|f'(x_0)|}\delta(x - x_0) \tag{6}$$

Using the distribution of Eq. (5) and the property (6) one can easily integrate over all concentrations:

$$P(I - I_0) = (I - I_0)^{-\gamma}\,\alpha \int \left(Ae^{\Delta G/RT}\right)^{\gamma-1} q(\Delta G) d(\Delta G) \tag{7}$$

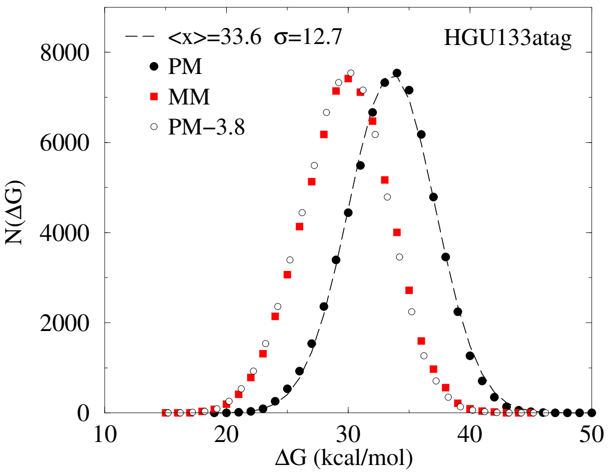

Figure 4: Distribution of hybridization free energy for the human chip set HG-U133. ΔG here is calculated from the nearest neighbor model, as discussed in the paper. The distributions are well approximated by a Gaussian (dashed line). Note also that PM and MM distributions are simply shifted by a constant free energy of 3.8 kcal/mol.

As the integrand does not depend on $I - I_0$ it merely provides a proportionality constant:

$$P(I - I_0) = \frac{\xi}{(I - I_0)^{-\gamma}} \tag{8}$$

Thus, the result of inferring a concentration probability distribution as in Eq. (5) is an intensity distribution that is also power law. This clear analytical result can be obtained only for such a distribution as in Eq. (5). Yet, once one knows $q(\Delta G)$ as in Fig. 4 and $P(I - I_0)$ from the experiment, in principle it is possible to find the corresponding $r(c)$, by numerical inversion of Eq. (4).

Anomalous histograms

For a comparison we present in Fig. 5 two anomalous *H. Sapiens* experiments. In these the background level is exceptionally high compared to all other experiments analyzed. Note that there is a split between PM and MM histograms around $I \simeq 10,000$. The Langscal package yields an average PM/MM ratio of $a \approx 1.1$ for these experiments, well below the average value for all other human chips analyzed. In Table 1 the average PM/MM ratio for all the flagged human experiments is shown.

Finally Fig. 6 is the scanned image of the *D. melanogaster* chip which performs badly in the boxplot shown in Fig. 4 of the main text.

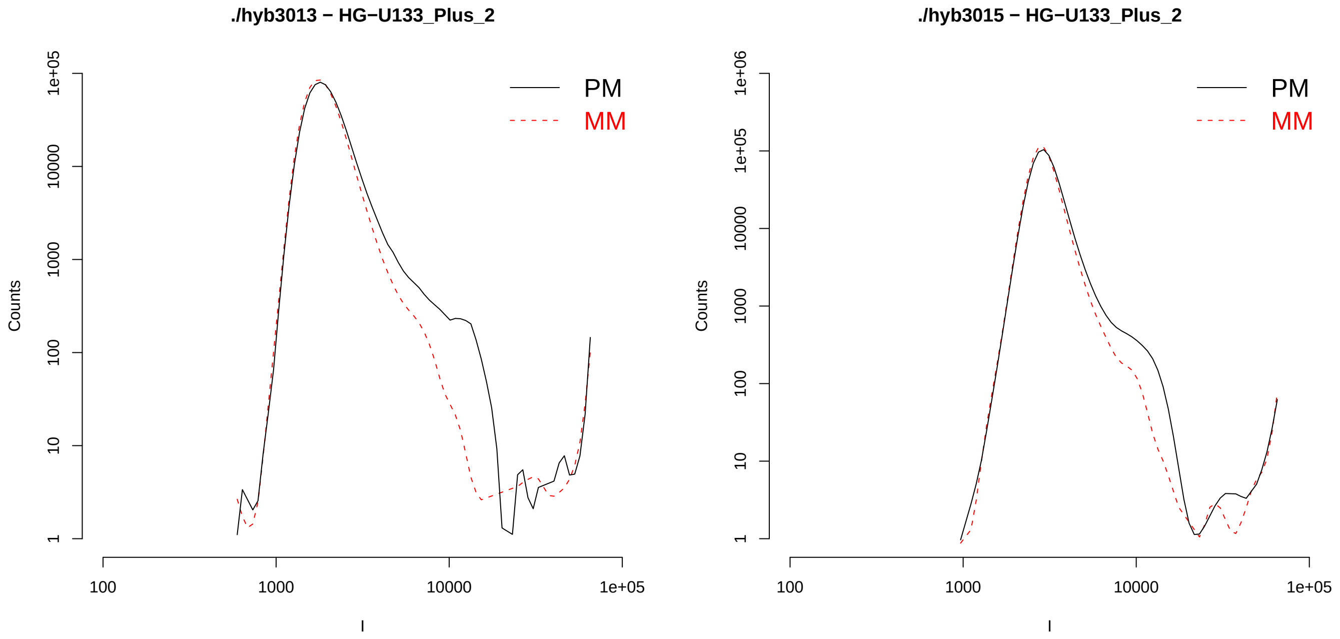

Figure 5: Global histograms for two HG-U133 chips also flagged as hybrizations of insufficient quality.

Hyb Id	Organism	Langmuir global a	SF	Affymetrix QC parameters Average background	Noise values
3013	*H. sapiens*	1.32	1.143	1272.14	68.84
3014	*H. sapiens*	1.12	0.919	2226.5	132.09
3015	*H. sapiens*	1.15	1.032	2040.62	143.11
3181	*H. sapiens*	1.27	1.086	1522.82	97.59

Table S 1: Four hybridizations on *H. sapiens* chips had a low global a value. All of them were flagged based on the Affymetrix guidelines. In particular, they had an average background far above the range, advised by Affymetrix (i.e., from 20 to 100), and noise values that exceed the typical values obtained with our scanner (ranging from 1 to 5).

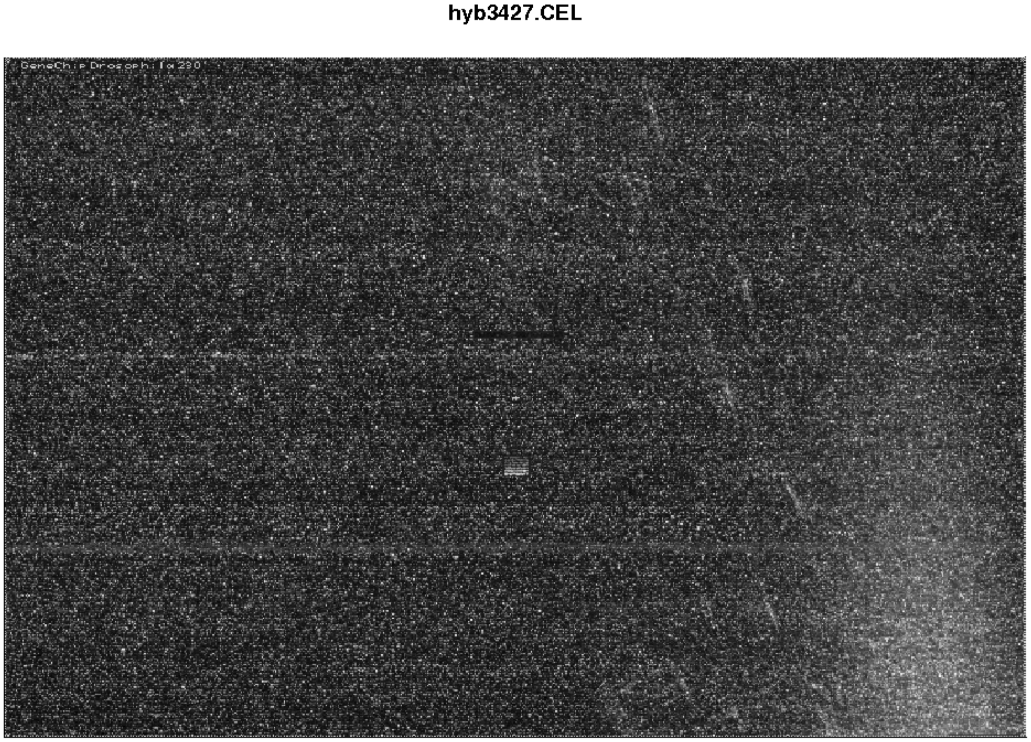

Figure 6: One *D. melanogaster* chip has a global a value around 3, i.e. lower than expected. This chip was already flagged as abnormal, as it has a stain in the bottom right corner.

Rescaled histograms for the remaining triplets of the *D. Melanogaster* experiment presented

Figures 7, 8, 9, 10, 11, 12 and 13 show the rescaling of background subtracted PM and MM histograms for all the 64 triplets in the D. Melanogaster experiment presented in the main paper. The dashed blue line represents the rescaled MM histogram.

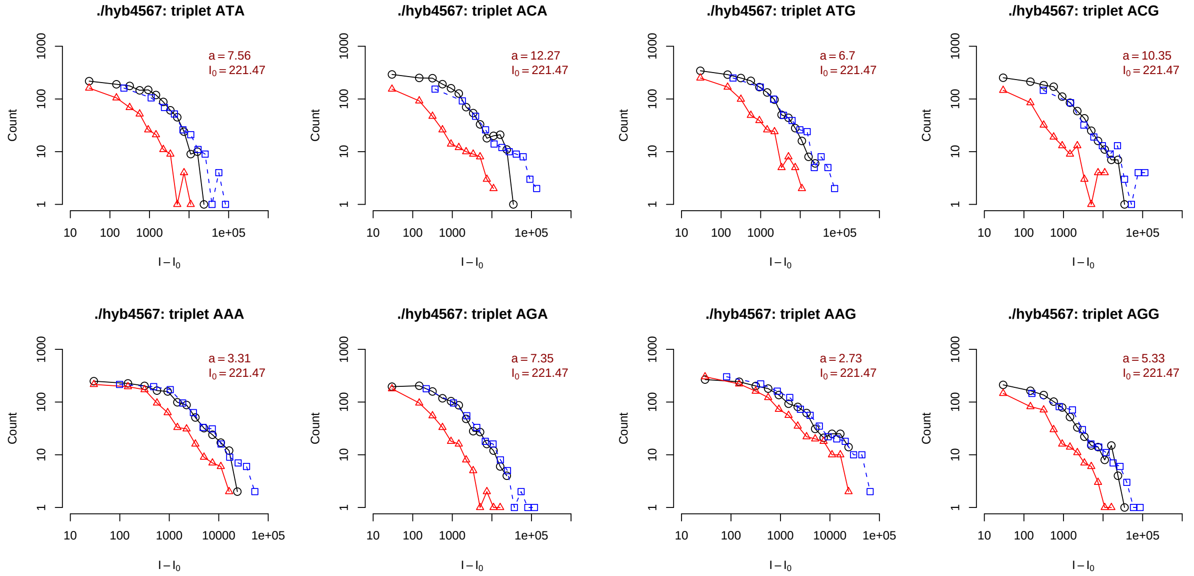

Figure 7: Histograms of the intensities of PMs (black circles) and MMs (red triangles) for the *D. melanogaster* shown in the article. 8 of the remaining 56 cases of the triplet rescalings are shown.

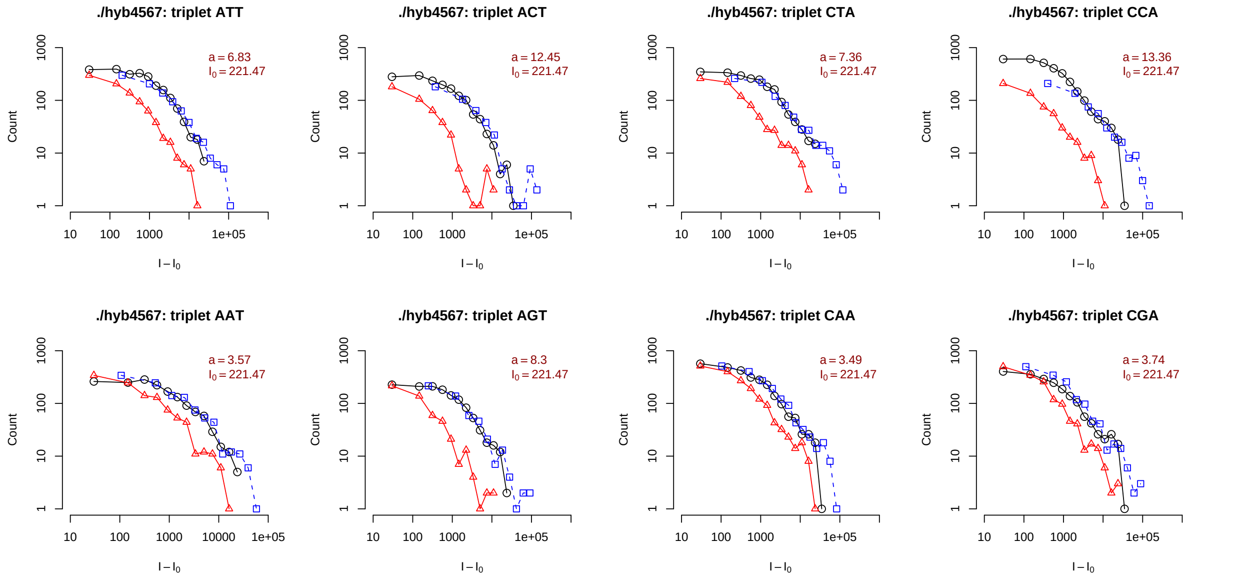

Figure 8: Histograms of the intensities of PMs (black circles) and MMs (red triangles) for the *D. melanogaster* shown in the article. 8 of the remaining 56 cases of the triplet rescalings are shown.

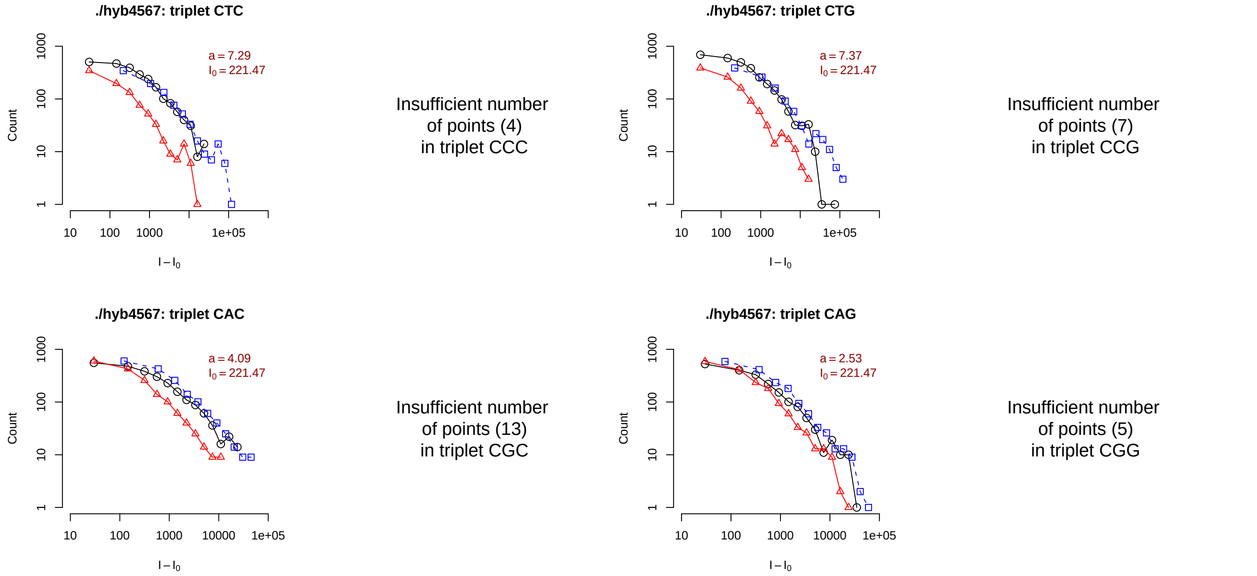

Figure 9: Histograms of the intensities of PMs (black circles) and MMs (red triangles) for the *D. melanogaster* shown in the article. 8 of the remaining 56 cases of the triplet rescalings are shown. Notice that Affymetrix seems to (almost) avoid sequences with some particular central triplets that have only Cytosine and Guanine, i.e. CCC, CGC, CCG, CGG, GCC, GGC, GCG and GGG.

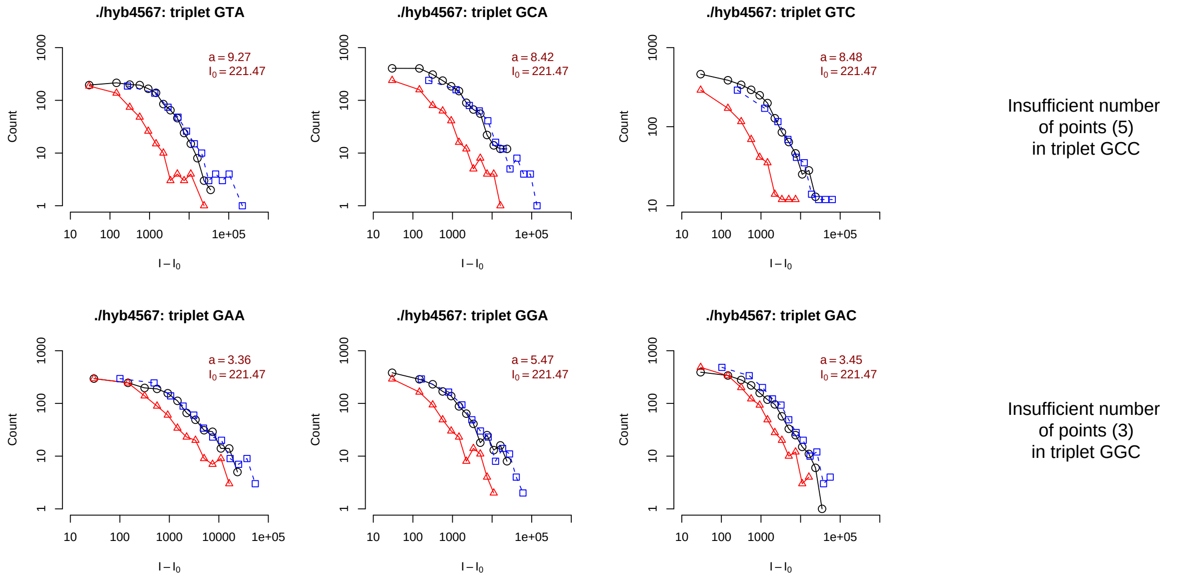

Figure 10: Histograms of the intensities of PMs (black circles) and MMs (red triangles) for the *D. melanogaster* shown in the article. 8 of the remaining 56 cases of the triplet rescalings are shown.

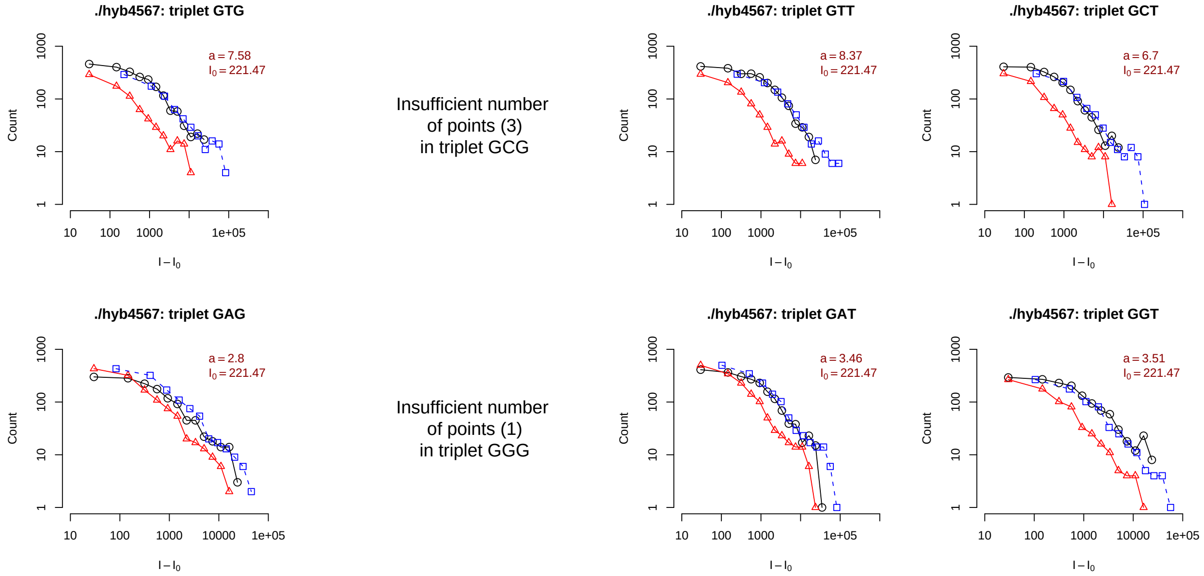

Figure 11: Histograms of the intensities of PMs (black circles) and MMs (red triangles) for the *D. melanogaster* shown in the article. 8 of the remaining 56 cases of the triplet rescalings are shown.

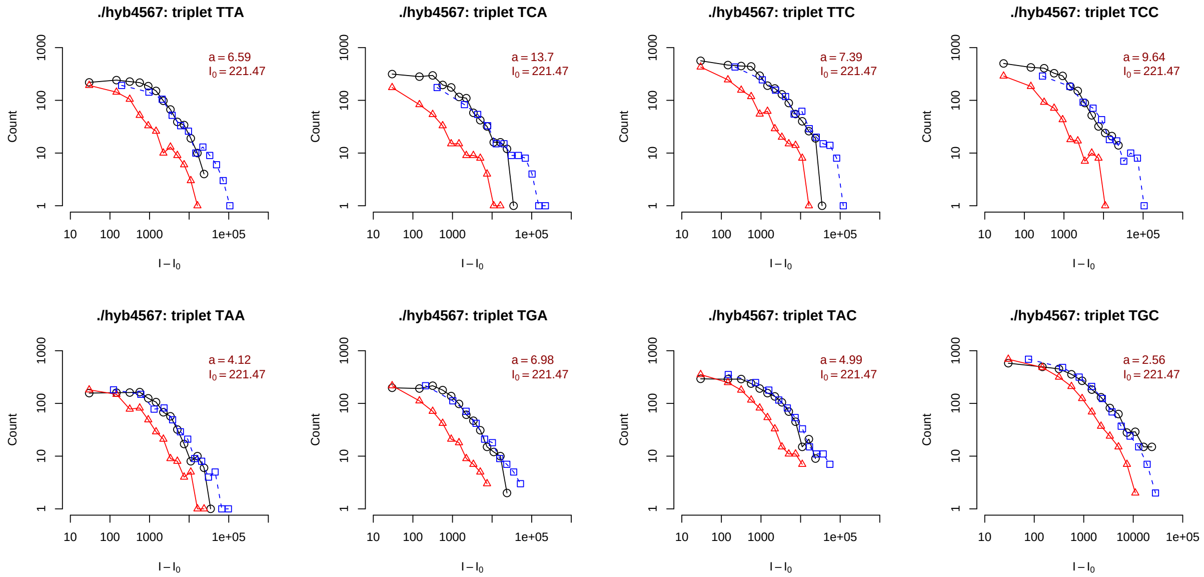

Figure 12: Histograms of the intensities of PMs (black circles) and MMs (red triangles) for the *D. melanogaster* shown in the article. 8 of the remaining 56 cases of the triplet rescalings are shown.

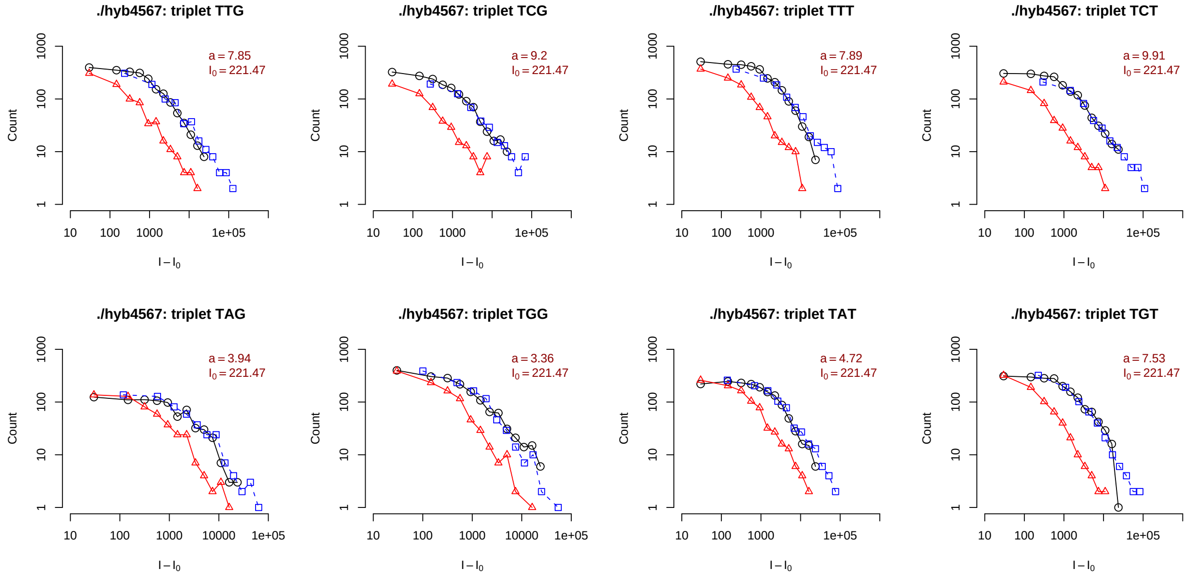

Figure 13: Histograms of the intensities of PMs (black circles) and MMs (red triangles) for the *D. melanogaster* shown in the article. 8 of the remaining 56 cases of the triplet rescalings are shown.

Triplet rescaling histograms: other organisms

In this section we present again a few histograms of the background subtracted histograms for PM and MM, the probes being grouped according to their central triplet. Data are now pertinent to different experiments on different organisms. Note that the distances between MM and PM histograms are always greater for the *A. thaliana* chip than the *H. sapiens* one. In particular some of the PM and MM histograms (look for example at AAT and AGT central triplets) seem to perform rather badly in the human chip.

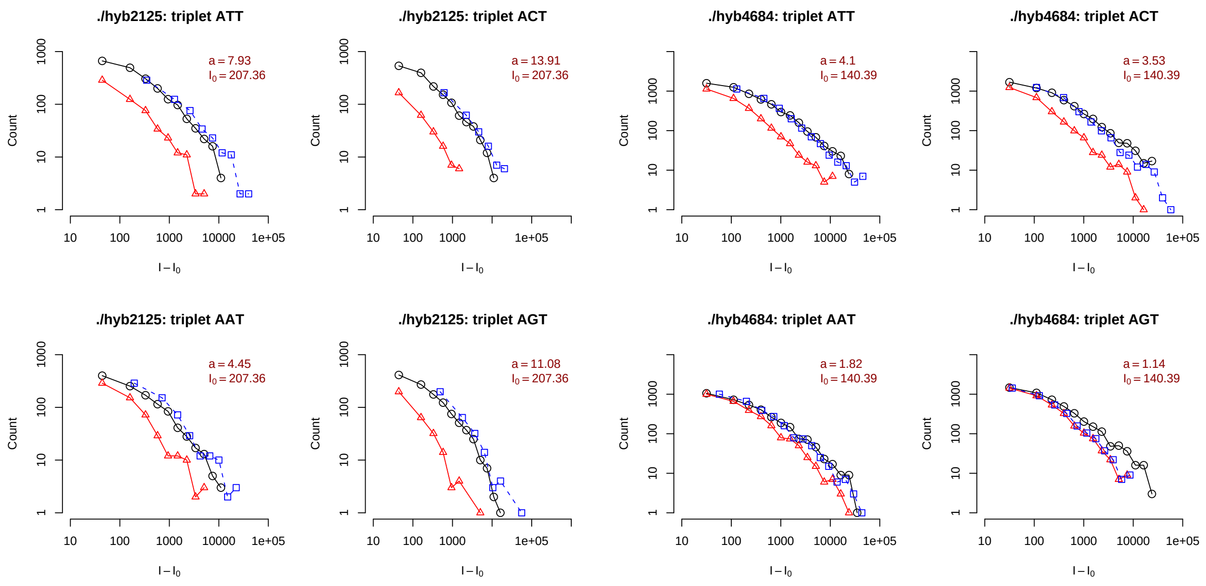

Figure 14: Scaling PM/MM ratio per triplet for two experiments on *A. thaliana* (left) and *H. sapiens* (right).

Supplementary plots for rescaled histograms: triplet parameters

Figures 15, 16 and 17 show an overview of the values of the 64 PM/MM ratios found grouping probes according to their central triplet. The triplets are once more grouped according to the mismatch. Notice the common trend approximatively independent of the organism, while quantitative accordance only holds when comparing experiments on the same organism. Moreover the inequality $a_{TU} < a_{AA} < a_{GG}$ ($a_{x\bar{x}}$ meaning the average value of the PM/MM ratios for the same mismatch) is consistent with what expected for hybridization in suolution.

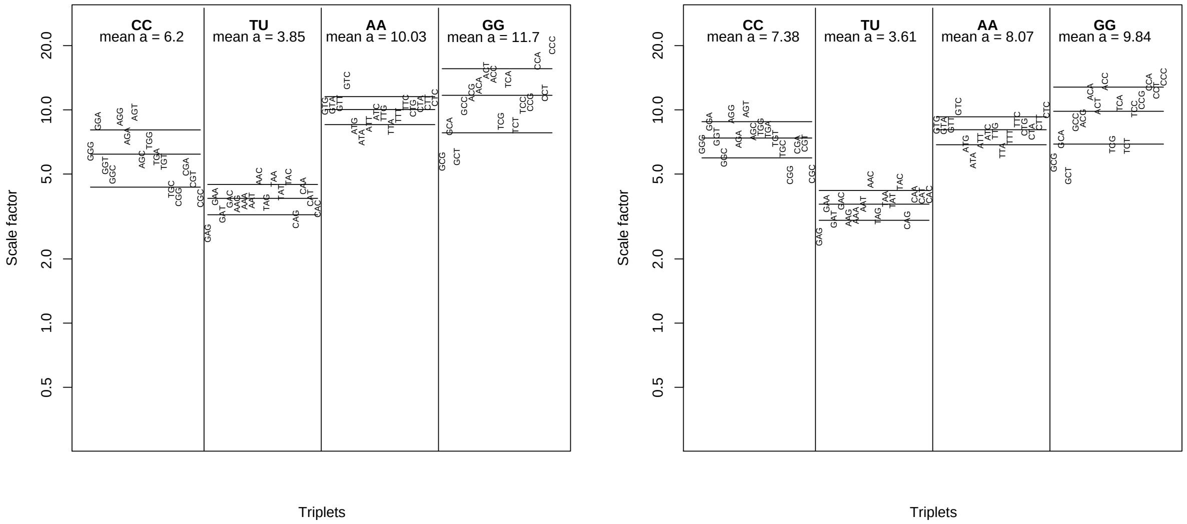

Figure 15: Scaling PM/MM ratio per triplet for two experiments on *A. thaliana*.

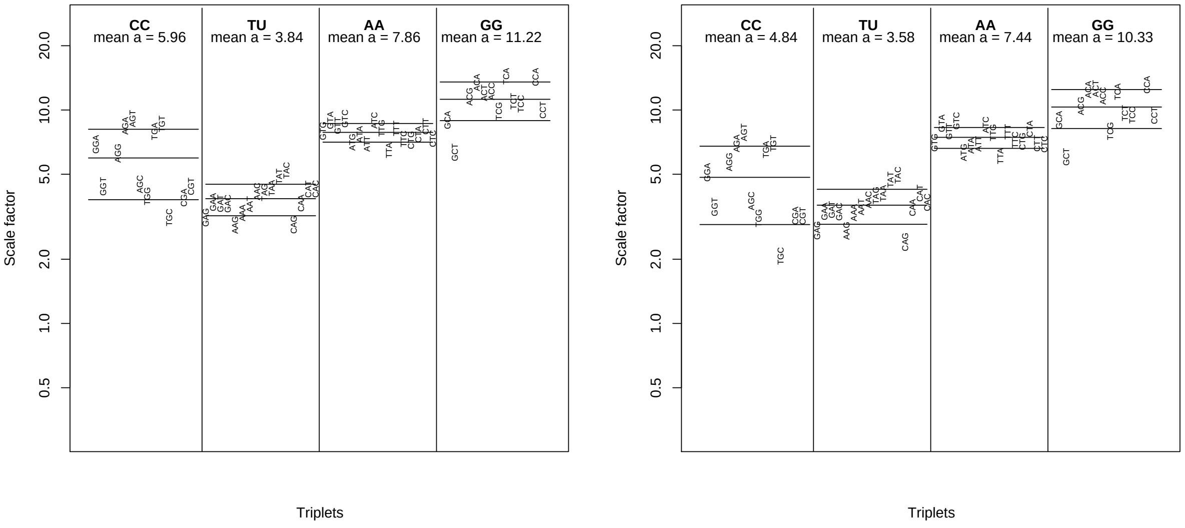

Figure 16: Scaling PM/MM ratio per triplet for experiments on *D. melanogaster*.

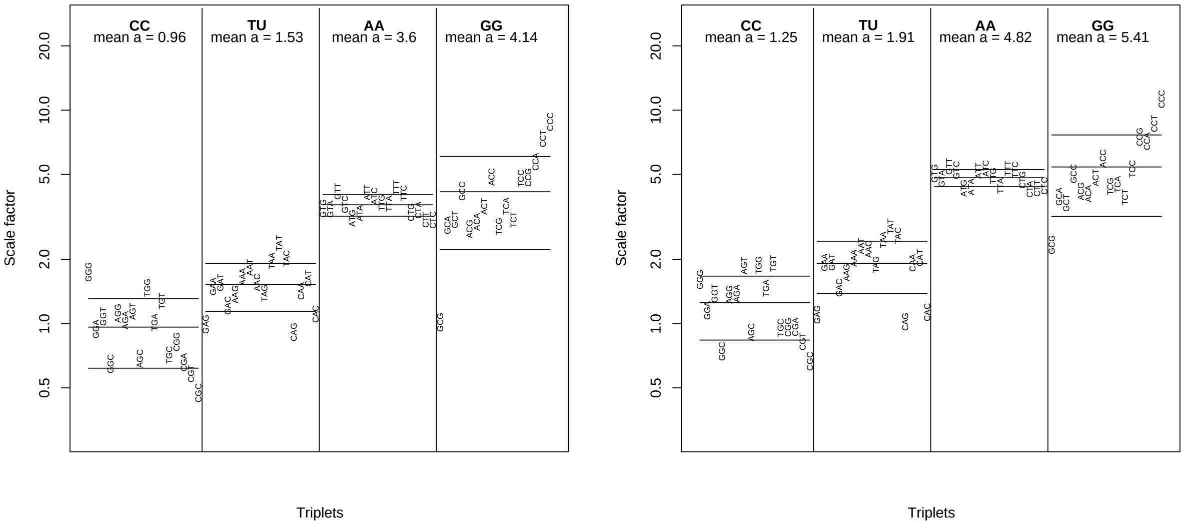

Figure 17: Scaling PM/MM ratio per triplet for experiments on *H. sapiens*.

Supplementary plots for global rescaling histograms

In this section a few other histograms of the background subtracted PM and MM are presented. This time all the probes on the chip are taken into account at once. The dashed blue line represents as usual the rescaled MM histogram. Note that the drop (clearly evident for PM histograms) is due to saturation effect, the denominator in Eq. (1) being no longer negligible.

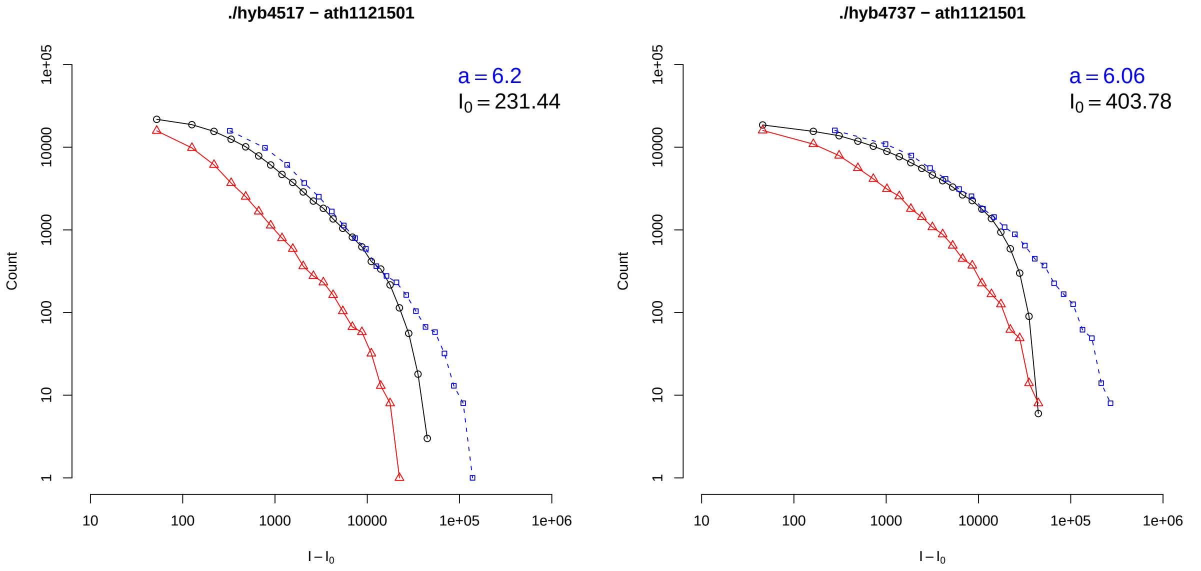

Figure 18: Histograms of the background-subtracted intensities of PMs (black circles) and MMs (red triangles) for two experiments on *A. thaliana*.

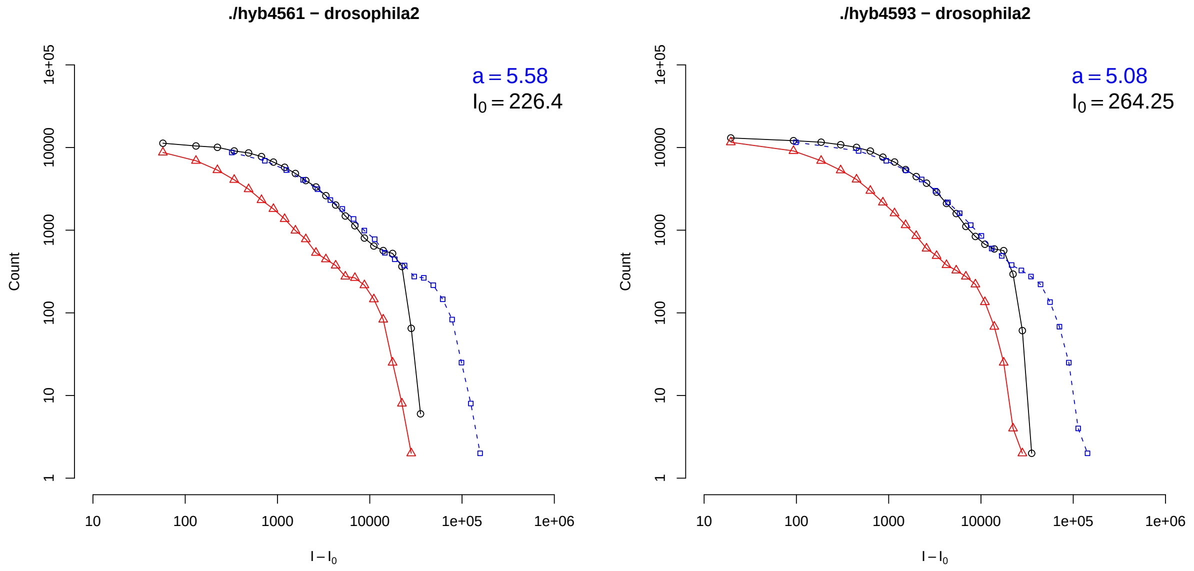

Figure 19: Histograms of the background-subtracted intensities of PMs (black circles) and MMs (red triangles) for experiments on *D. melanogaster*.

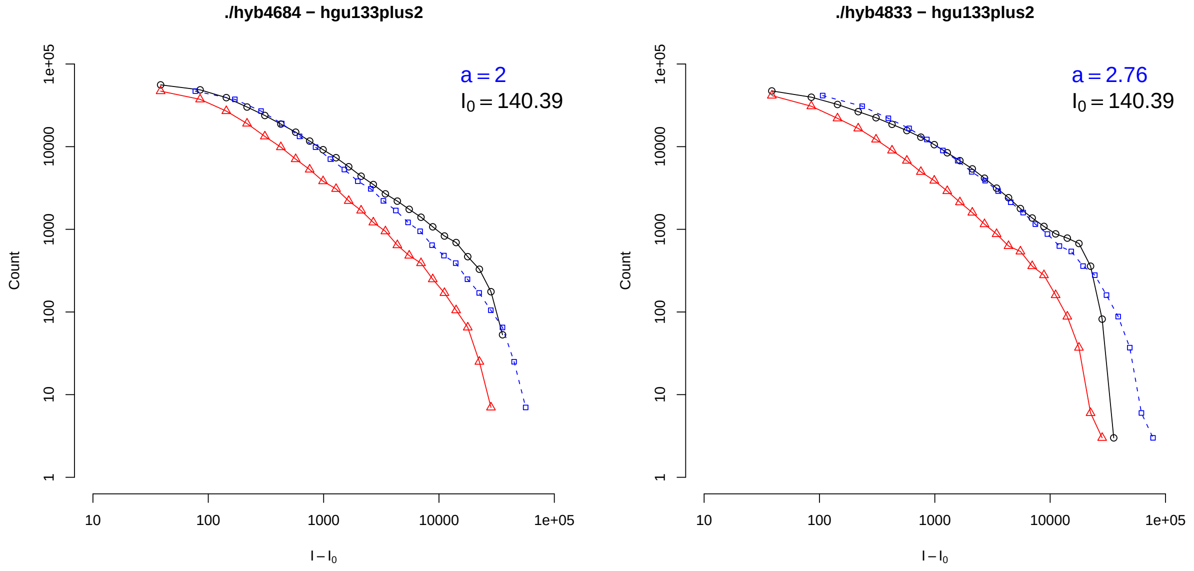

Figure 20: Histograms of the background-subtracted intensities of PMs (black circles) and MMs (red triangles) for experiments on *H. sapiens*.
